# *Crithmum maritimum* Extract Restores Lipid Homeostasis and Metabolic Profile of Liver Cancer Cells to a Normal Phenotype

**DOI:** 10.1007/s11130-024-01188-5

**Published:** 2024-05-06

**Authors:** Davide Gnocchi, Dragana Nikolic, Rosa Rita Paparella, Carlo Sabbà, Antonio Mazzocca

**Affiliations:** Interdisciplinary Department of Medicine, University of Bari School of Medicine, Piazza G. Cesare, 11 - 70124 Bari, Italy

**Keywords:** *Crithmum maritimum* L., Edible wild plant, Hepatocellular carcinoma, Lipid-lowering activity, Metabolic health-promoting activity

## Abstract

Hepatocellular carcinoma (HCC) is an alarming epidemiological clinical problem worldwide. Pharmacological approaches currently available do not provide adequate responses due to poor effectiveness, high toxicity, and serious side effects. Our previous studies have shown that the wild edible plant *Crithmum maritimum* L. inhibits the growth of liver cancer cells and promotes liver cell differentiation by reducing lactic acid fermentation (Warburg effect). Here, we aimed to further characterise the effects of *C. maritimum* on lipid metabolism and markers of cellular metabolic health, such as AMP-activated protein kinase (AMPK), Sirtuin 1 (SIRT1), and Sirtuin 3 (SIRT3), as well as the insulin signalling pathway. To better mimic the biological spectrum of HCC, we employed four HCC cell lines with different degrees of tumorigenicity and lactic acid fermentation/Warburg phenotype. Lipid accumulation was assessed by Oil Red O (ORO) staining, while gene expression was measured by real-time quantitative PCR (RT-qPCR). The activation of AMPK and insulin signalling pathways was determined by Western blotting. Results indicate that *C. maritimum* prevents lipid accumulation, downregulates lipid and cholesterol biosynthesis, and modulates markers of metabolic health, such as AMPK, SIRT1 and SIRT3. This modulation is different amongst HCC cell lines, revealing an important functional versatility of *C. maritimum*. Taken together, our findings corroborate the importance of *C. maritimum* as a valuable nutraceutical, reinforcing its role for the improvement of metabolic health.

**Supplementary Information:**

The online version contains supplementary material available at 10.1007/s11130-024-01188-5.

## Introduction

The alarming rise in metabolic abnormalities currently underlies many diseases, including cancer, cardiovascular disease, and neurodegenerative disorders [1–4]. Plant-derived products have gained renewed interest in several areas of medicine, including oncology [5], and plant-based diets and nutritional supplements are growing in importance [6]. Hepatocellular carcinoma (HCC) is the sixth leading cause of cancer death worldwide and is projected to become the third leading cause of cancer death in Western countries by 2030 [7]. Metabolic dysregulation is now considered an important driver of HCC, as many HCC cases are the final stage of pathological progression from hepatic lipid accumulation (steatosis) [8] to inflammation, fibrosis and ultimately development of HCC [9]. In fact, it is estimated that approximately 35% of HCC cases may develop directly from simple steatosis [10]. The main strategy for HCC treatment is surgery, while pharmacological approaches rely on a combination of tyrosine kinase inhibitors and immunotherapy [11]. However, these interventions have not met expectations from the perspective of effectiveness and toxicity. In this context, there is an urgent need to scientifically demonstrate the effectiveness of new, less toxic, and more effective specific approaches to prevent and treat HCC. Based on this assumption, phytonutrients and nutritional supplements can provide valuable support. [12]. *C maritimum* has been known to people since ancient times. People living along the Mediterranean coast use seaweed as a diuretic, purifier, digestive aid, antiscorbutic, anti-cold and anti-inflammatory, as well as a wound healer and anthelmintic [13]. *C. maritimum* can also be used as a food: its leaves can be eaten in salads, cooked, or treated with vinegar like capers. This typical Apulian preparation is included in the list of traditional agricultural products of the Ministry of Agriculture [14]. Further information and references on these topics as well as on the botanical, phytochemical, and ethnopharmacological characteristics of *C. maritimum* can be found in the Supplementary Materials. Despite its long tradition as a food and medicinal plant, the scientific basis behind the efficacy of *C. maritimum* is missing, and its nutraceutical properties as an anti-tumour agent are lacking in evidence. Moreover, although in recent years several scientific works dealt with the antitumor action of plant extracts, we have been the first to focus on *C. maritimum* and to the characterisation of its “systemic-metabolic” effects (Additional References on this issue can be found in the Supplementary Material). We were the first to demonstrate the cytostatic effect of the ethyl acetate extract of *C. maritimum* in HCC [15], linking it to a “rescheduling” of the tumour “metabolic signature,” with decreased levels of several metabolites, such as lactate, amino acids, cholesterol and polyunsaturated fatty acids (PUFA), as well as choline and phosphocholine, which fuel tumour growth. Additionally, we found an increase in healthy monounsaturated fatty acids (MUFAs) [16]. Our previous studies also showed that *C. maritimum* promoted oxidative phosphorylation (OXPHOS) in HCC cells [17] and could be used in combination with tyrosine kinase inhibitors to reduce their doses and thus reduce toxicity [18]. We later found that *C. maritimum* sensitises HCC cells to tyrosine kinase inhibitors by inhibiting lactate fermentation and rewires the cancerous metabolic profile of HCC, promoting a phenotype more similar to normal hepatocytes [19]. Here, we focused on further characterising the nutraceutical properties of *C. maritimum* on the regulation of the metabolic phenotype of HCC, using four cell lines with different degree of aggressiveness and differentiation state, and then with a different metabolic phenotype, ranging from prevalent oxidative phosphorylation (OXPHOS) to prevalent, lactic acid fermentation/Warburg effect [20]. This broad metabolic diversity was chosen to better characterise the impact of *C. maritimum* on the biological and metabolic changes found in liver metabolic diseases and HCC. Hence, the aim of this study was to provide preclinical evidence on the role of *C. maritimum* as a solid supportive nutraceutical tool in the clinical management of HCC, by the evaluation of the effect on key metabolic pathways which are dysregulated in HCC. Our results indicate that *C. maritimum* has potent homeostatic effects on the dysregulated metabolism of HCC cells by: i) decreasing the expression of the genes controlling lipogenesis and cholesterogenesis, ii) activating AMP-activated protein kinase (AMPK), Sirtuin 1 (SIRT1) and Sirtuin 3 (SIRT3), which are well-known metabolic health markers, iii) improving insulin response related signalling. As these metabolic pathways are dysregulated in HCC, *C. maritimum* has great potential as a nutraceutical tool for adjuvant therapy and prevention of HCC.

## Materials and Methods

### Preparation and Characterisation of C. Maritimum Ethyl Acetate Extract

*C. maritimum* ethyl acetate extract was prepared and characterised as we previously reported [15]. The ethyl acetate fraction was the most biologically active if compared to fractions obtained with polar solvents (ethanol) and with other non-polar solvents (Hexane, methanol) [15, 16]. Cytotoxicity of ethyl acetate extract and IC_50_ has been determined in our previous work [15].

### Cell Lines and Culturing

A detailed description can be found in the Supplementary Materials section. Briefly, cells were treated with 0.5 mM *C. maritimum* ethyl acetate extract, as we previously showed that this represents the optimal dose in terms of effects and cytotoxicity in HCC cell lines [15–17, 19, 21].

### Oil Red O (ORO) Staining for Evaluation of Lipid Accumulation

A detailed description can be found in the Supplementary Materials section.

### Quantitative Real-Time PCR (RT q-PCR)

A detailed description can be found in the Supplementary Materials section.

### Immunoblotting Analyses

A detailed description can be found in the Supplementary Materials section.

### Statistical Analyses

A detailed description can be found in the Supplementary Materials section.

## Results

**Fig. 1:**
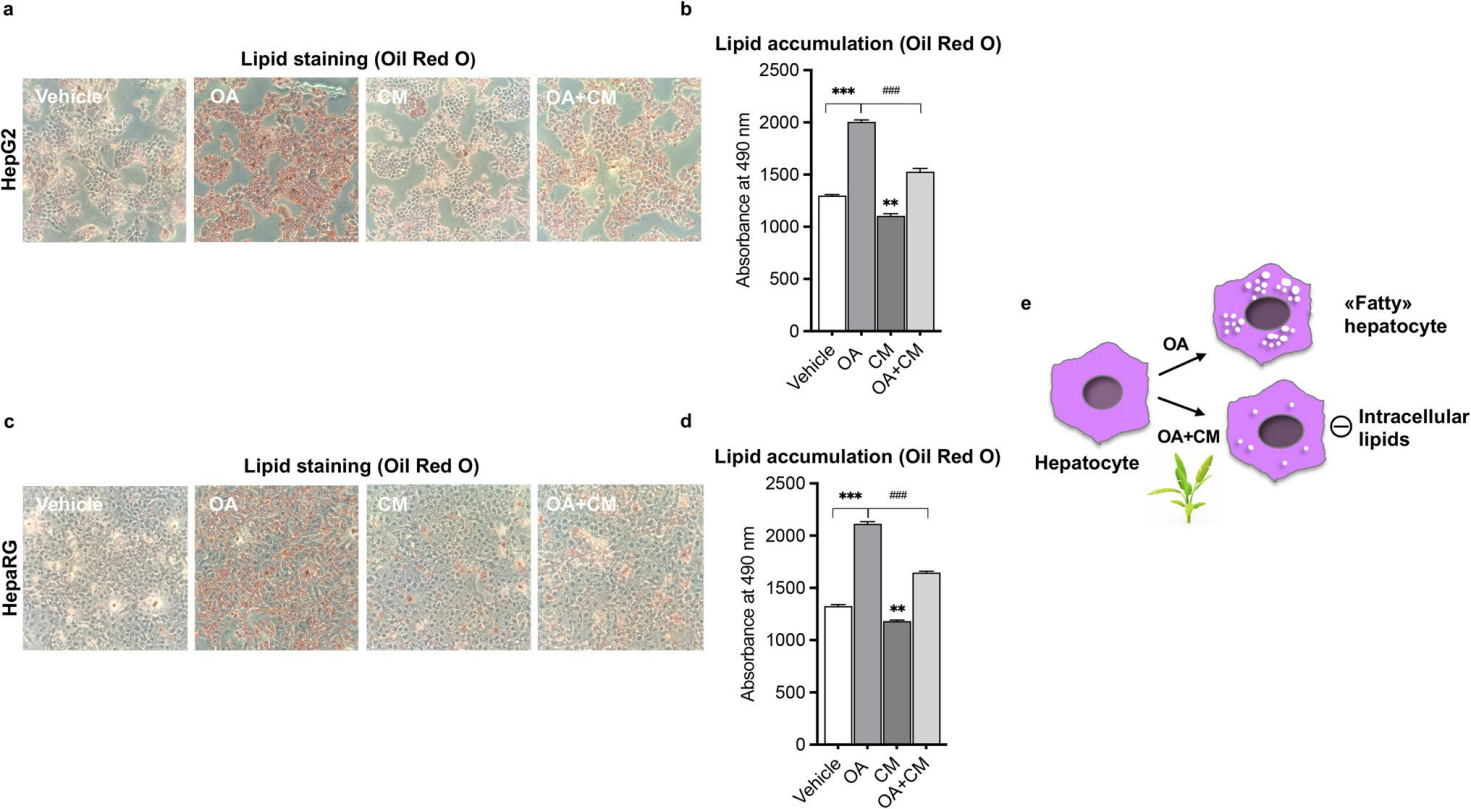
*C. maritimum* prevents lipid accumulation in HCC cell lines. Cells were treated for 24 h with 100 μM oleic acid (OA), *C. maritimum* 0.5 mM ethyl acetate extract (CM), and OA + CM. Lipid accumulation was evaluated by Oil Red O (ORO) staining. ORO pictures and quantification in HepG2 (**a**-**b**) and HepaRG cells (**c**-**d**). **e** Graphical summary of the key message described in the Figure. *** *p* < 0.001 compared to Vehicle; ** *p* < 0.01 compared to Vehicle ### *p* < 0.001 compared to OA

First, we explored the role of *C. maritimum* in regulating lipid and metabolic health homeostasis in HCC. Figure 1 shows that 0.5 mM ethyl acetate extract of *C. maritimum* prevented lipid accumulation in two HCC cell lines, HepG2 and HepaRG. These two cell lines were chosen because they have been shown to be good models of hepatic lipid accumulation in vitro [22]. To assess lipid accumulation, we used Oil Red O (ORO) staining, which stains neutral lipids [9]. In detail, Fig. 1a, b show that *C. maritimum* (CM) prevented the formation of intracellular lipid droplets induced by 24 h treatment with 100 μM oleic acid (OA) as determined by ORO staining. In fact, treatment with CM determined a significative inhibition of lipid accumulation (about 75%).

**Fig. 2:**
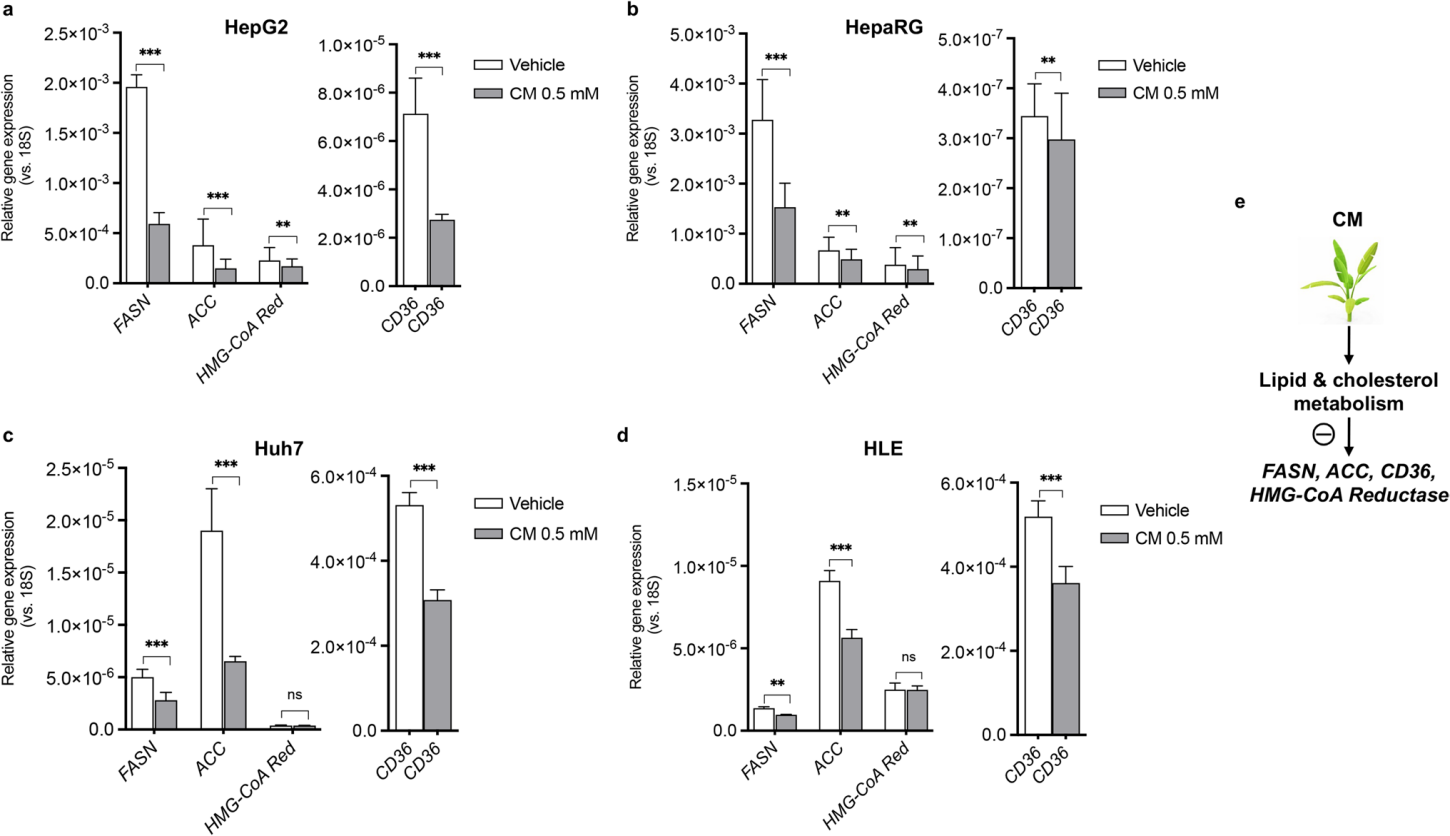
*C. maritimum* restores the expression of lipid metabolism genes in HCC cell lines. Cells were treated with *C. maritimum* 0.5 mM ethyl acetate extract (CM) for 24 h. The expression of four key genes in lipid and cholesterol metabolism in four HCC cell lines, HepG2 (**a**), HepaRG (**b**), Huh7 (**c**), and HLE (**d**), was measured by RT q-PCR. *** *p* < *0.001*, ** *p* < 0.01, ns = not significant compared with vehicle

Next, we analysed the expression of two major genes involved in the lipid biosynthesis pathway, fatty acid synthase (FASN) and acetyl-CoA carboxylase (ACC). Overexpression of these two genes has been associated to hepatocarcinogenesis [23, 24]. We also considered the expression of HMG-CoA reductase (HMG-CoA Red), a key gene involved in cholesterol biosynthesis, whose overexpression has been associated with the development of HCC [25]. Finally, we evaluated the expression of CD36, a fatty acid transporter whose overexpression has been associated with the development of hepatic steatosis and HCC [26]. Results show that *C. maritimum* effectively counteracts lipid gene dysregulation in HCC cell lines (Fig. 2), which promotes the development of HCC [23–26]. In fact, all of the above-mentioned genes were significantly down-regulated by a 24 h treatment with *C. maritimum* in HepG2 and HepaRG, while no significant effect was observed for *HMG-CoA Red* in Huh7 and HLE. Taken together, these results suggest that *C. maritimum* can contribute to normalise the transformed lipid phenotype in well-established HCC.

**Fig. 3:**
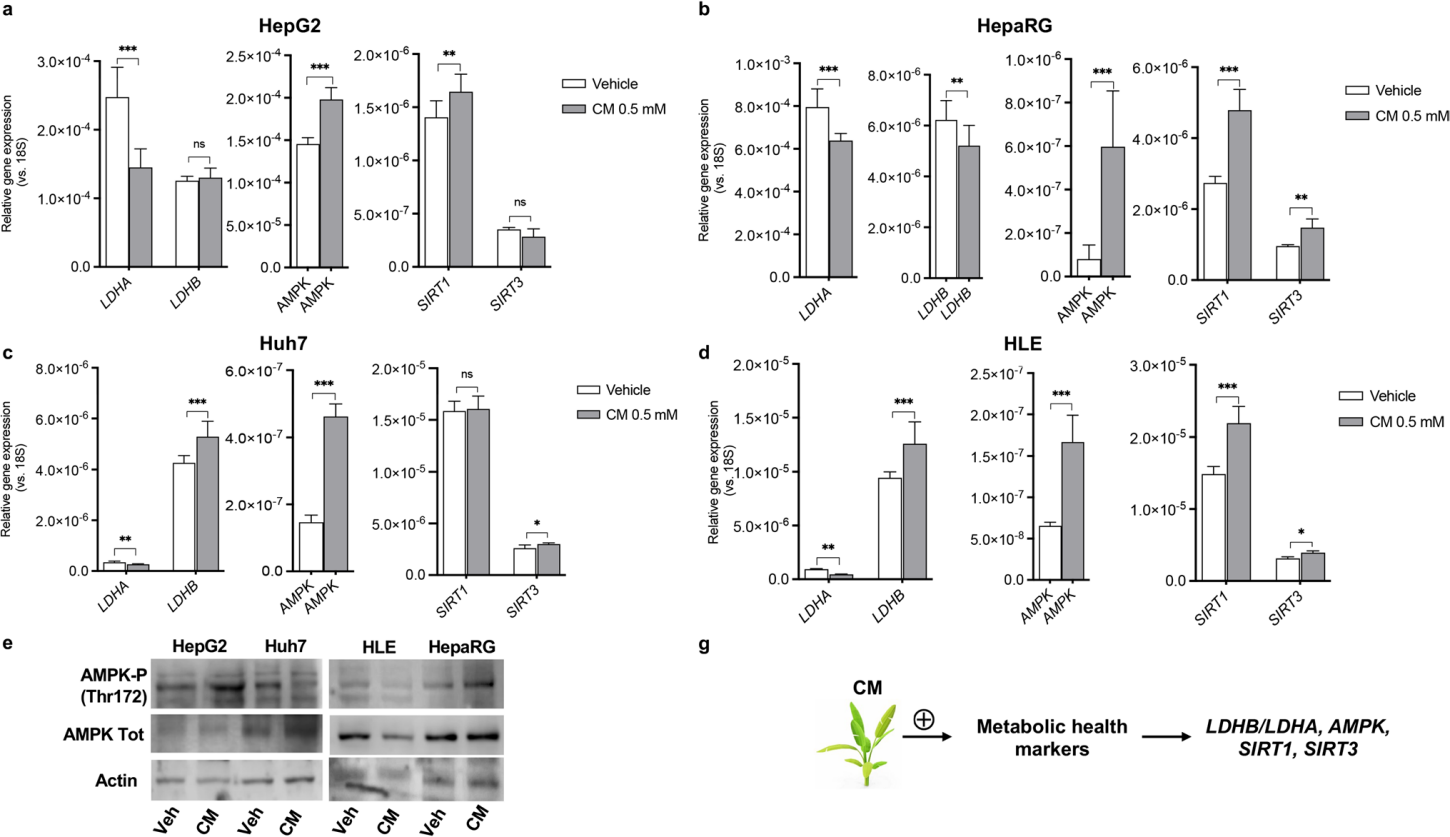
*C. maritimum* improves the expression of metabolic health marker genes in HCC cell lines. Cells were treated for 24 h with *C. maritimum* 0.5 mM ethyl acetate extract (CM). The expression of key genes involved in the control of metabolic health status was evaluated by RT q-PCR in four HCC cell lines: HepG2 (**a**), HepaRG (**b**), Huh7 (**c**), HLE (**d**). The activation of AMPK has been analysed by Western Blot in HepG2 and Huh7 cells (**e**). **f** Graphical summary of the key message described in the Figure. *** *p* < 0.001, ** *p* < 0.01, * *p* < 0.05, ns = not significant compared with vehicle

We then evaluated the nutritional potential of *C. maritimum* in modulating the expression of genes that are indicators of the metabolic state of the cell: lactate dehydrogenase A (LDHA), Lactate Dehydrogenase B (LDHB), AMP-activated protein kinase (*AMPK*), Sirtuin 1 (*SIRT1*), Sirtuin 3 (*SIRT3*). Each of these genes controls key steps in regulating metabolic homeostasis, and their expression is dysregulated in tumour cells and HCC, with *LDHA* typically upregulated [27] and *LDHB* downregulated [28]. All the other above-mentioned genes are downregulated [29]. Activation of *AMPK*, *SIRT1*, and *SIRT3* is believed to be a promising option for the prevention and treatment of HCC [30–32]. The data reported in Fig. 3 demonstrate that *C. maritimum* reverses the pathological expression of these genes (Fig. 3a-d). Overall, *C. maritimum* was effective against all four cell lines used, which are models of different degrees of HCC transformation, differentiation, and invasiveness. Compared with Huh7, HLE are less differentiated and more dependent on lactic acid fermentation [20], while Huh7 is more invasive and fermentative compared with HepG2 and HepaRG, which are less invasive and tumorigenic. HepaRG are considered a good model of non-transformed hepatocytes. It is noteworthy that, although there are some differences, *C. maritimum* remains effective regardless of the characteristics of the cell line used: The effects on LDHA and AMPK were significant in all four cell lines. *LDHB* expression increased in Huh7 and HLE, the two more aggressive and fermentative cell lines, but did not significantly change in HepG2, and it is slightly but significantly decreased in HepaRG*. SIRT1* expression was significantly increased in three cell lines: in HepG2, HepaRG, and HLE, but not in Huh7. *SIRT3* was significantly upregulated in three cell lines (HepaRG, Huh7, HLE), whereas no changes were measured in HepG2. Western Blot analysis was employed to demonstrate the activation of AMPK signalling by phosphorylation at Thr172 (Fig. 3e). Interestingly, a stronger activation of AMPK was observed in less aggressive cell lines (HepG2, HepaRG). Figure 3f summarises the results.

**Fig. 4:**
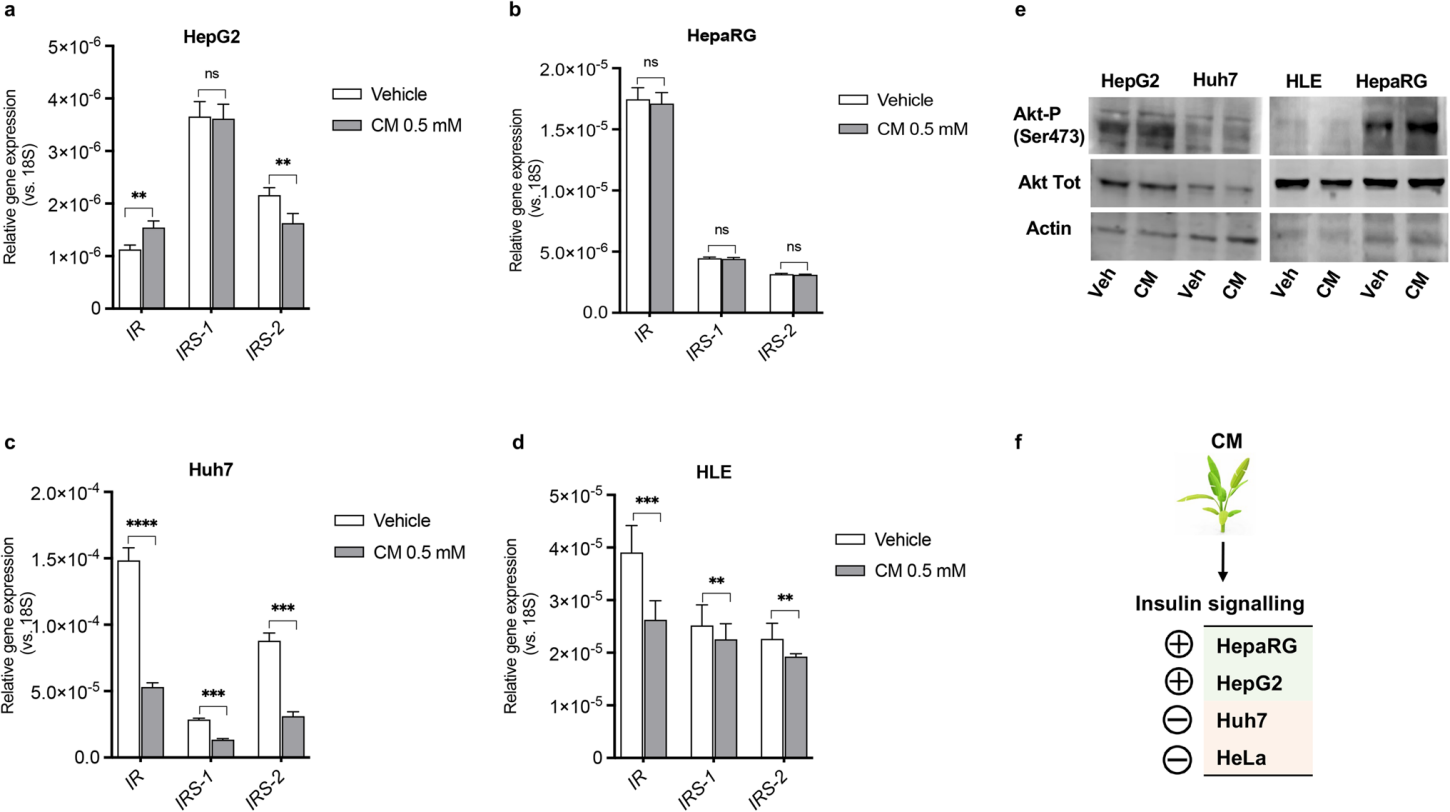
*C. maritimum* differentially regulates insulin signalling depending on the degree of tumorigenicity of HCC cells. For gene expression experiments, cells were treated for 24 h with *C. maritimum* 0.5 mM ethyl acetate extract (CM). The expression of three genes involved in the control of insulin signalling was evaluated by RT q-PCR in four HCC cell lines HepG2 (**a**), HepaRG (**b**), Huh7 (**c**), HLE (**d**). For Western Blot experiments, cells were treated for 45 min with *C. maritimum* 0.5 mM ethyl acetate extract (CM). Activation of insulin signalling has been verified by Western Blot by evaluating Akt phosphorylation at Ser473 in HepG2 and Huh7 cells (**e**). **f** Graphical summary of the information presented in the Figure. **** *p* < 0.0001, *** *p* < 0.001, ** *p* < 0.01, * *p* < 0.05, ns = not significant compared with vehicle

Finally, we investigated the role of *C. maritimum* in regulating the insulin signalling pathway, as its dysregulation has been implicated in the development and progression of HCC. In particular, we have measured the expression of the three main genes involved in the control of the pathway: Insulin Receptor (*IR*), Insulin Receptor Substrate 1 (*IRS-1*) and Insulin Receptor Substrate 2 (*IRS-2*). In addition, we evaluated the phosphorylation of Akt at Ser473, a well-established marker of activation of the insulin signalling pathway. The results reported in Fig. 4 paint an interesting scientific picture: indeed, *C. maritimum* showed different differential effects that varied according to the degree of invasiveness and Warburg phenotype of the cell lines used. In fact, in HepG2 and HepaRG, with a low tumorigenic/ “normal phenotype”, gene expression and Western blot analyses show an activation of insulin signalling: slight variations of *IR*, *IRS-1* and *IRS-2* gene expression (Fig. 4 a, e), while the phosphorylation of Akt at Ser473 was increased (Fig. 4b). In the more tumorigenic and invasive Huh7 and HLE cells, *C. maritimum* instead significantly downregulated the expression of the analysed insulin signalling genes, and no effect was observed on Akt phosphorylation at Ser473 (Fig. 4 c-e). Notably, *IRS-1* and *IRS-2* are upregulated in HCC and actively support the tumour phenotype [33]. A graphical summary is showed in Fig. 4f.

## Discussion

**Fig. 5:**
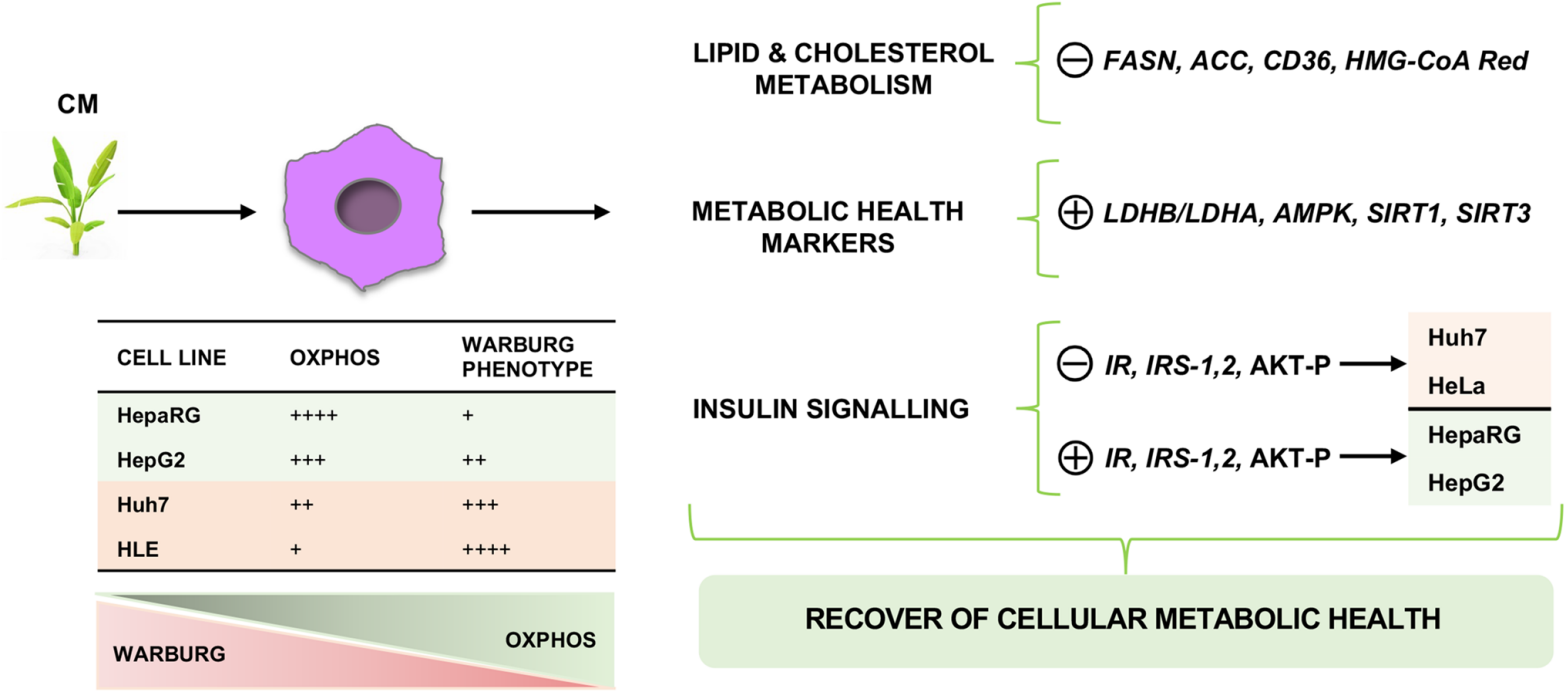
Schematic representation of the effects of *C. maritimum* on HCC cell lines with different degrees of tumorigenicity and Warburg phenotype. As described in the text, *C. maritimum* prevents lipid accumulation, reduces the expression of key genes that control lipogenesis, and selectively promotes the normalisation of metabolic markers of cellular health. Specifically, insulin signalling was inhibited in the highly tumorigenic/Warburg-dependent cell lines Huh7 and HLE, whereas it was promoted in the low tumorigenic/OXPHOS-dependent cell lines HepaRG and HepG2

Hepatocellular carcinoma (HCC) is now an epidemiologic emergency, which can be explained by the increase in metabolic diseases. We previously reported that *C. maritimum* inhibits HCC cell growth [15] and modulates metabolic [16] and bioenergetic characteristics of HCC cells by inhibiting lactic acid fermentation and activating OXPHOS [17]. We also demonstrated that *C. maritimum* reduced the expression of HCC markers *α-fetoprotein* (*α-FP*) and *α1-antitrypsin* (*α1-AT*) and improved the sensitivity of HCC cell to anti-neoplastic drugs by determining a shift in the HCC bioenergetic profile from lactic acid fermentation to OXPHOS [19]. Interestingly, in line with this, we recently showed that OXPHOS impairment is associated with drug resistance in HCC cell lines [34]. This supports the possible applicability of *C. maritimum* as a concrete nutraceutical option in the clinical management of HCC. Our current findings provide evidence that *C. maritimum* interacts with HCC cells to improve their lipid and metabolic profiles and to orient them towards the phenotype of non-transformed cells. Moreover, here, we have demonstrated the “normalisation” of three well-known “cell metabolic health promoters,” such as *AMPK*, *SIRT1* and *SIRT3*, as well as the selective inhibition of insulin signalling only in the more aggressive cell lines. This reinforces the importance of *C. maritimum* as a valuable nutritional option supporting a lifestyle aimed at preventing and treating metabolic disorders, which are becoming a serious health problem worldwide, especially in Western countries [1–4]. Overall, *C. maritimum* shows a strong multifaceted bioactive action, which can be an important resource in the management of HCC and possibly of other types of tumours. It is worth to underline here the systemic and multi-targeted action exerted by the blend of compounds found in *C. maritimum* (Fig. S1), which guarantees a full-spectrum action on several of the central bioenergetic traits characteristic of transformed hepatocytes. It is also important to emphasize that the effects of *C. maritimum* appear to be regulated according to the cellular metabolic phenotype. In fact, *C. maritimum* stimulated insulin signalling in low tumorigenic cells (mainly relying on OXPHOS), whereas in high tumorigenic cells (Warburg phenotype) an inhibition of insulin signalling was observed (Fig. 5). This important characteristic gives to *C. maritimum* specificity and selectivity towards normal or HCC cells.

All these versatile characteristics make *C. maritimum* particularly important in the context of HCC therapy and prevention of the predisposing dysmetabolic liver conditions, even if compared with other efficacious plant-based approaches, such as Chinese herbal formulation YIV-906, which have been recently tested in clinical trials as a supplement to improve conventional HCC drug treatments [35]. In the next step of our study, we will continue to explore the OXPHOS-mediated promotion of drug sensitivity and the mechanism behind the drug sensitization of *C. maritimum.* This may disclose additional translational applications for the nutraceutical properties of *C. maritimum*.

Future research activities will also be directed towards the preparation of formulations for use in human clinical trials, case–control studies in HCC patients, and combining appropriate extract preparations with standard therapy. We are also about to begin a clinical study to test the effects of a *C. maritimum* formulation as a nutritional supplement to improve metabolic health, with a focus on triglyceride and cholesterol levels and insulin sensitivity.

## Conclusions

Based on our previous findings and those presented here, we propose C. maritimum as a specific nutraceutical tool for developing strategies to support conventional HCC drug treatment and reduce side effects. Additionally, supplementation with *C. maritimum* may be used to prevent metabolic diseases that cause or contribute to abnormal liver metabolism and ultimately hepatocellular carcinoma. Our study demonstrated that *C. maritimum* has broad-spectrum and multi-target effects, which may provide its application in the treatment of other types of cancer.

### Supplementary Information

Below is the link to the electronic supplementary material.

Supplementary file1 (DOCX 671 KB)

Supplementary file2 (PDF 253 KB)

## Data Availability

All data presented in this study are available upon request.
