# Supplementary material for: Crithmum maritimum Extract Restores Lipid Homeostasis and Metabolic Profile of Liver Cancer Cells to a Normal Phenotype
Source: Plant Foods Hum Nutr. 2024 May 6;79(2):417–24. doi: 10.1007/s11130-024-01188-5 (PMC11178603; doi:10.1007/s11130-024-01188-5)
Supplement: Supplementary file 1 — Supplementary file1 (DOCX 671 KB) [file 11130_2024_1188_MOESM1_ESM.docx]

***Manuscript – Supplementary Material***

***Crithmum maritimum* extract restores lipid homeostasis and metabolic profile of liver cancer cells to a normal phenotype**

Davide Gnocchi^1^, Dragana Nikolic^1^, Rita Rosa Paparella^1^, Carlo Sabbà^1^, Antonio Mazzocca^1^

^1^ Interdisciplinary Department of Medicine, University of Bari School of Medicine,

Piazza G. Cesare, 11 - 70124 Bari, Italy

**Correspondence:**

Antonio Mazzocca, M.D., Ph.D., Interdisciplinary Department of Medicine, University of Bari School of Medicine, Piazza G. Cesare, 11 I-70124 Bari, Italy

Tel.: +39 080 5593593

Email: [antonio.mazzocca@uniba.it](mailto:antonio.mazzocca@uniba.it)

**Materials and Methods**

*Cell lines and culturing*

HepG2, Huh7 and HLE cell lines were purchased from the JCRB cell bank [cat. # JCRB1371, cat. # JCRB0404, cat. # JCRB1054]. HepG2 and Huh7 were grown in Dulbecco's modified Eagle's medium (DMEM) with 1 g/L glucose, 4 mM glutamine, and 1 mM sodium pyruvate [Corning cat. #10-014-CVR], supplemented with 10% Foetal Bovine Serum (FBS) [Corning cat. # 35-079-CV], 1X MEM-Nonessential Amino Acids [Corning cat. # 25-025-CIR], 20 mM Hepes Buffer [Aurogene cat. # AU-L0180-500], 1X Antibiotic-Antimycotic solution [Corning cat. # 30-004-CI]. HLE was grown in RPMI [Corning cat. # -CVR] supplemented with 10% FBS, 1X MEM-Nonessential Amino Acids, 20 mM Hepes Buffer, 1X Antibiotic-Antimycotic solution. HepaRG cell line was obtained through a Material Transfer Agreement from Bio-Predic International. HepaRG was grown in Williams E medium [Sigma-Aldrich cat. # W1878] supplemented with 10% FBS, 5 μg/mL insulin [Sigma-Aldrich cat. #I9278], 50 μM hydrocortisone succinate. Cells were kept under standard conditions (humidified atmosphere, 37°C and 5% CO_2_).

HepG2 and HepaRG have been treated with oleic acid [Sigma-Aldrich cat. # O1383] using volumes not exceeding 1% volume of the cell culture media. Treatment with *Crithmum maritimum* L. extract has been performed with volumes not exceeding 1% volume of the cell culture media. Ethanol and DMSO respectively were used as vehicle control at 0.5% or 1% volume of cell culture media. Cells were treated 24 h after plating for all of the experiments reported.

*Oil Red O (ORO) staining*

Cells were initially fixed with paraformaldehyde 4% for at least 1 h at room temperature. After two washes with PBS, cells were incubated with ORO solution for a minimum of 1 h at room temperature. After two PBS washes, pictures have been taken using a Nikon TMS (model DS-Fi1) associated with a “Nikon Digital Sight” digital camera and with “NIS-Elements F3.0” software. A quantitative assessment of the staining was performed by eluting the colour with isopropanol and reading absorbance at λ=510 nm.

For haematoxylin counterstaining, after ORO staining, cells were washed twice with PBS and once with distilled water, and then incubated with haematoxylin for 5 minutes. After two water washes, cells were imaged.

*Quantitative Real-Time PCR (q-RT-PCR)*

RNA was extracted using the “Aurum Total RNA Mini Kit" [Bio-Rad cat. #732-6820] following the producer's directions of use. The amount and the quality of the extracted RNA were determined by using an “IMPLEN N50” Nanophotometer. 1 μg RNA was used for cDNA synthesis, which has been performed with the “iScript Advanced cDNA Synthesis Kit” [Bio-Rad cat. #1725038]. q-RT-PCR was performed using a “SsoAdvanced Universal SYBR Green Supermix” using 500 nM primers. Fluorescence was detected using an “Applied Biosystems 7300 Real-Time PCR System”. Primer sequences are available upon request from the corresponding author.

*Immunoblotting analyses*

Cell lysates were made with Cell Signaling lysis buffer [Cell Signaling cat. #9803] added with protease and phosphatase inhibitors [Roche cat. #04693159001 and 04906837001]. Samples were prepared with Laemmli Sample Buffer [BioRad cat. #161-0737] complemented with 5% β-mercaptoethanol. Proteins were separated by SDS-PAGE and then blotted onto nitrocellulose membranes. Primary antibody incubation was performed overnight at 4°C with gentle shaking using the antibodies reported below. Membranes were developed using Licor c-Digit. Each panel of the reported blots was obtained by stripping the same membrane two times and re-probing it with the indicated antibodies.

Antibodies employed: Phospho-AMPKα (Thr172) Antibody [Cell Signaling cat. #2531]; AMPKα Antibody [Cell Signaling cat. #2532]; Phospho-Akt (Ser473) Rabbit mAb [Cell Signaling cat. #4058]; Akt Antibody [Cell Signaling cat. #9272]; Actin [Santa Cruz cat. # sc-7210]; Secondary antibody: Anti-rabbit IgG, HRP-linked [Cell Signaling cat. #7074].

*Statistical analyses*

The normality of data was verified with D'Agostino-Pearson's Omnibus K2 test. One-Way ANOVA followed by Dunnett's post-hoc test was used to determine statistical significance when data were normally distributed.

**Supplementary Figures**

**Fig. S1** Graphical representation of the major metabolites found in *Crithmum maritimum* L. used in this study [see also Gnocchi D et al., Sci Rep. 2021 Jan 13;11(1):1259. PMID: 33441568].

**Supplementary Text**

**Botanical classification and geographical distribution**

*Crithmum maritimum* L., the only species of the genus *Crithmum*, is a perennial perennial herb in the *Apiaceae* or *Umbelliferae* family. The name derives from the Greek word “Cretzmon,” probably due to the leaves’ shape or to the similarity of seed shape with that of barley [1,2]. *C. maritimum* has many folk names. In English the most common are: “rock samphire, samphire, marine fennel, crest marine.” In France, *C. maritimum* is known as “perce-pierre, passepierre, fenouil marine, criste-marine.” In Germany it is is known as “Seefenchel, Meerfenchel” and in Spain as “perejil marino, hinojo marino”. In Italy, *C. maritimum* has different designations in different regions of the country: “finocchio marino, erba di S. Pietro, cretamo, critama, bacicci, basiggia and spaccasassi” [3]. Botanically, C. maritimum is classified as a perennial halophyte and its habitat is the coastline, where it grows on or between rocks, seawalls, and beaches.

Regarding geographical distribution, *C. maritimum* is endemic to North Africa, the Mediterranean countries, up to France, the British Isles, and Ireland. It has also been reported along the Pacific coast[4,2]. *C. maritimum* plants plants have a branched, bushy appearance and grow to a maximum height of about 60 cm. The leaves are small and succulent, the flowers develop at the beginning of summer (July) and last until the end of summer (September), while the fruits are usually produced in October-November and the fruit ripening period lasts until December [1,2].

**Traditional and present use of *Crithmum maritimum***

***Historical perspective***

*C. maritimum* has been known since ancient times. It is mentioned in Hippocrates “Corpus Hippocraticum” as well as in Plinius "Historia Naturalis," where it is suggested as a good remedy for renal conditions and gout [5]: "The Critmo, is highly praised by Hippocrates. It is one of those wild herbs that are eaten and it is certainly this, that according to Callímaco, is served by the farmer Ecale to Theseus." In the Byzantine period, it is described with the name "crithmom" in the "Dynameron", a medical manuscript written by Nikolaos Myrepsos [6]. Of note, *Crithmum maritimum* is also mentioned in Shakespeare's "King Lear" (Act 4, scene 6): “it is terrible job… The habit of climbing the cliffs to collect it because often halfway, those who search for it, fall”…

***Traditional medicinal and food uses***

Populations living along the Mediterranean coast are classified as *C. maritimum* many medicinal properties: It has diuretic, purifying, digestive, anti-scurvy, anti-cold, anti-inflammatory, wound-healing and anthelmintic properties [2]. Additionally, fishermen used *C. maritimum* leaves to treat scurvy, and Italian tradition holds that *C. maritimum* infusions improve digestion, while decoctions can treat prostatitis and cystitis [2]. In Spain, the pickled leaves are still believed to help improve digestion and have a diuretic effect [7]. In the Easter Riviera of Liguria, Italy, a decoction of *C. maritimum* is used as an aid to liver function [8], while in southern Italy (Amalfi Coast), it is used to relieve symptoms of colds and coughs [9]. *C. maritimum* essential oil has also been shown to have antibacterial properties [10] and antimycotic [11] action. Notably, insecticidal effects of *C. maritimum* against insects of different genera have been reported [12-14].

*C. maritimum* is also appreciated as a food: its leaves can be eaten in salads, cooked, or treated with vinegar like capers. This preparation in Apulia is included in the Ministry of Agriculture list of traditional agri-food products [4]. In the United Kingdom, leaves and stems of *C. maritimum* are cooked along with pickled cucumbers and capers to obtain the “Rock Samphire hash” [4].

**Phytochemical characteristics**

The phytochemical characterisation of *C. maritimum*, which has been performed during the last decades, has provided some scientific support to the above-described folk medicine traditions and usages.

The leaves of *C. maritimum* are rich in Vitamin C, flavonoids, carotenoids, and in several classes of bioactive compounds. Furthermore, edible oil can be extracted from its seeds, which is considered a good source of essential fatty acids [15]. As for bioactive compounds, the reported quantification varies among studies. This is probably due to the different protocols employed, as well as to the growing area and the period of the year [16]. Indeed, the phenolic composition and antioxidant activity of *C. maritimum* were different between the vegetative and flowering physiological stages. Interestingly, the antioxidant properties were significantly higher in the vegetative stage compared to the flowering stage, while the amount of phenolic compounds and flavonoids did not significantly differ between the two physiological stages [17]. Regarding phenolic compounds, some studies reported that *C. maritimum* is rich in chlorogenic acid, quinic acid, and caffeoylquinic acid. The concentrations of these compounds are higher in leaves compared to flowers and stems [18,19]. It has been reported that several volatile compounds are also commonly found in young shoots and fruits, most of which are terpenoids [2].

The metabolite content of the dried powder of *C. maritimum* employed in our studies was determined by ^1^H-NMR metabolomics. The powder was particularly rich in chlorogenic acid, whereas the ethyl acetate extract consisted mainly of falcarindiol, flavonoids, carotenoids and hydroxycinnamates, and hydroxybenzoic acid derivatives (Table 1).

**Table 1**

From “Gnocchi D. et al., Plant Food Hum Nutr. (2022) (PMID: 35831770)” [20]

**Supplementary References**

1. Atia A, Chokri H, Mokded R, Barhoumi Z, Abdelly C, Smaoui A (2011) Anatomy of the fruit of the halophyte Crithmum maritimum L. with emphasis on the endosperm structure and histochemistry. Afr J Biotechnol 10 (45):9193-9199

2. Atia A, Barhoumi Z, Mokded R, Abdelly C, Smaoui A (2011) Environmental eco-physiology and economical potential of the halophyte Crithmum maritimum L. (Apiaceae). J Med Plants Res 5 (16):3564-3571

3. Renna M (2018) Reviewing the Prospects of Sea Fennel (Crithmum maritimum L.) as Emerging Vegetable Crop. Plants (Basel) 7 (4). doi:10.3390/plants7040092

4. Renna M, Gonnella M, Caretto S, Mita G, Serio F (2017) Sea fennel (Crithmum maritimum L.): from underutilized crop to new dried product for food use. Genet Resour Crop Ev 64 (1):205-216. doi:10.1007/s10722-016-0472-2

5. Aliotta G, Pollio A (1994) Useful plants in renal therapy according to Pliny the Elder. Am J Nephrol 14 (4-6):399-341. doi:10.1159/000168755

6. Coiffard L, Piron-Frenet M, Amicel L (1993) Geographical variations of the constituents of the essential oil of Crithmum maritimum L., Apiaceae. Int J Cosmet Sci 15 (1):15-21. doi:10.1111/j.1467-2494.1993.tb00064.x

7. Carrio E, Valles J (2012) Ethnobotany of medicinal plants used in Eastern Mallorca (Balearic Islands, Mediterranean Sea). J Ethnopharmacol 141 (3):1021-1040. doi:10.1016/j.jep.2012.03.049

8. Cornara L, La Rocca A, Marsili S, Mariotti MG (2009) Traditional uses of plants in the Eastern Riviera (Liguria, Italy). J Ethnopharmacol 125 (1):16-30. doi:10.1016/j.jep.2009.06.021

9. Savo V, Giulia C, Maria GP, David R (2011) Folk phytotherapy of the Amalfi Coast (Campania, Southern Italy). J Ethnopharmacol 135 (2):376-392. doi:10.1016/j.jep.2011.03.027

10. Senatore F, Napolitano F, Ozcan M (2000) Composition and antibacterial activity of the essential oil from Crithmum maritimum L. (Apiaceae) growing wild in Turkey. Flavour Frag J 15 (3):186-189. doi:Doi 10.1002/1099-1026(200005/06)15:3<186::Aid-Ffj889>3.0.Co;2-I

11. Glamoclija J, Sokovic M, Grubisic D, Vukojevic J, Milinekovic I, Ristic M (2009) Antifungal activity of Critmum maritimum essential oil and its components against mushroom pathogen Mycogone perniciosa. Chem Nat Compd+ 45 (1):96-97. doi:10.1007/s10600-009-9242-0

12. Tsoukatou M, Tsitsimpikou C, Vagias C, Roussis V (2001) Chemical intra-Mediterranean variation and insecticidal activity of Crithmum maritimum. Z Naturforsch C 56 (3-4):211-215

13. Polatoglu K, Karakoc OC, Yucel YY, Gucel S, Demirci B, Baser KHC, Demirci F (2016) Insecticidal activity of edible Crithmum maritimum L. essential oil against Coleopteran and Lepidopteran insects. Ind Crop Prod 89:383-389. doi:10.1016/j.indcrop.2016.05.032

14. Pavela R, Maggi F, Lupidi G, Cianfaglione K, Dauvergne X, Bruno M, Benelli G (2017) Efficacy of sea fennel (Crithmum maritimum L., Apiaceae) essential oils against Culex quinquefasciatus Say and Spodoptera littoralis (Boisd.). Ind Crop Prod 109:603-610. doi:10.1016/j.indcrop.2017.09.013

15. Ben Hamed K, Ben Youssef N, Ranieri A, Zarrouk M, Abdelly C (2005) Changes in content and fatty acid profiles of total lipids and sulfolipids in the halophyte Crithmum maritimum under salt stress. Journal of Plant Physiology 162 (5):599-602. doi:10.1016/j.jplph.2004.11.010

16. Mekinic IG, Simat V, Ljubenkov I, Burcul F, Grga M, Mihajlovski M, Loncar R, Katalinic V, Skroza D (2018) Influence of the vegetation period on sea fennel, Crithmum maritimum L. (Apiaceae), phenolic composition, antioxidant and anticholinesterase activities. Ind Crop Prod 124:947-953. doi:10.1016/j.indcrop.2018.08.080

17. Jallali I, Megdiche W, M'Hamdi B, Oueslati S, Smaoui A, Abdelly C, Ksouri R (2012) Changes in phenolic composition and antioxidant activities of the edible halophyte Crithmum maritimum L. with physiological stage and extraction method. Acta Physiol Plant 34 (4):1451-1459. doi:10.1007/s11738-012-0943-9

18. Nabet N, Boudries H, Chougui N, Loupassaki S, Souagui S, Burlo F, Hernandez F, Carbonell-Barrachina AA, Madani K, Larbat R (2017) Biological activities and secondary compound composition from Crithmum maritimum aerial parts. Int J Food Prop 20 (8):1843-1855. doi:10.1080/10942912.2016.1222541

19. Meot-Duros L, Magne C (2009) Antioxidant activity and phenol content of Crithmum maritimum L. leaves. Plant Physiol Biochem 47 (1):37-41. doi:10.1016/j.plaphy.2008.09.006

20. Gnocchi D, Sabba C, Mazzocca A (2022) The Edible Plant Crithmum maritimum Shows Nutraceutical Properties by Targeting Energy Metabolism in Hepatic Cancer. Plant Foods Hum Nutr 77 (3):481-483. doi:10.1007/s11130-022-00986-z
