# Supplementary material for: Crithmum maritimum Extract Restores Lipid Homeostasis and Metabolic Profile of Liver Cancer Cells to a Normal Phenotype
Source: Plant Foods Hum Nutr. 2024 May 6;79(2):417–24. doi: 10.1007/s11130-024-01188-5 (PMC11178603; doi:10.1007/s11130-024-01188-5)
Supplement: Supplementary file 2 — Supplementary file2 (PDF 253 KB) [file 11130_2024_1188_MOESM2_ESM.pdf]

HepG2 Huh7

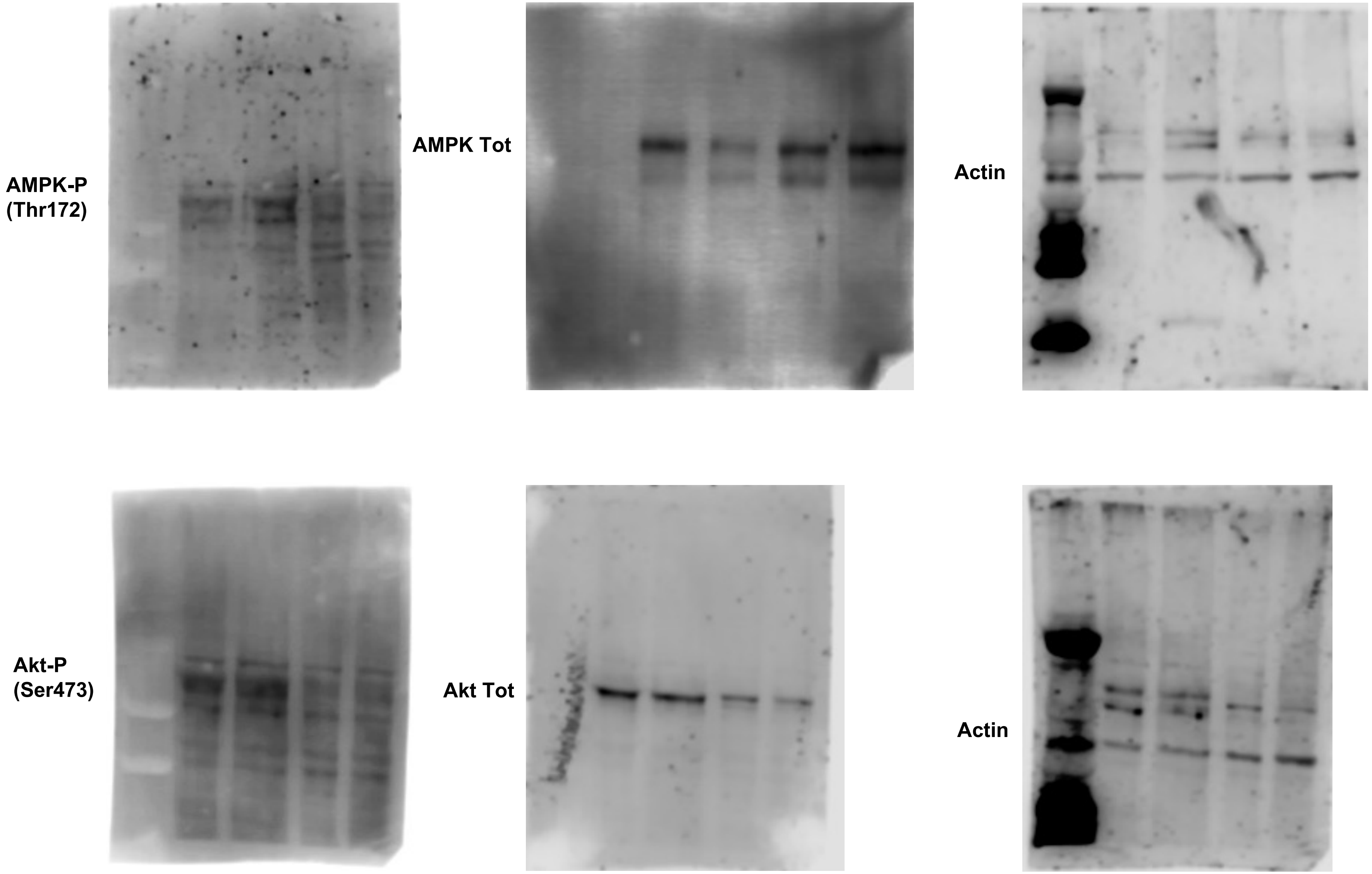

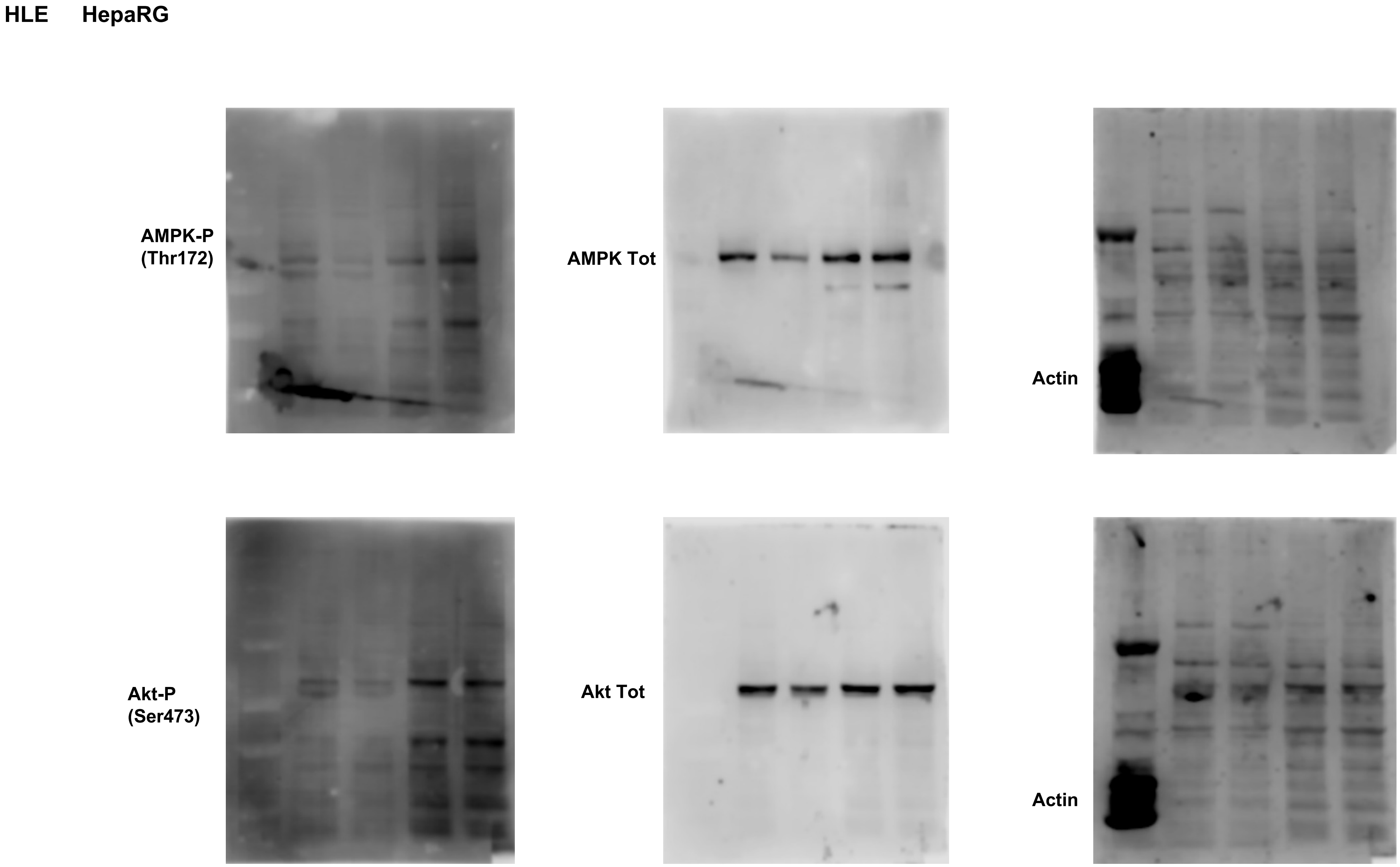
HLE
HepaRG
AMPK-P
(Thr172)
AMPK Tot
Actin
Akt-P
(Ser473)
Akt Tot
Actin
